# Intravitreal anti‐vascular endothelial growth factor injections and risks of stroke in patients with neovascular age‐related macular degeneration—A registry‐based cohort study

**DOI:** 10.1111/aos.17534

**Published:** 2025-06-07

**Authors:** A. H. Falemban, K. Söderberg‐Löfdal, F. Jonsson, S. Almlöf‐Sarman, M. von Euler, I. Westborg

**Affiliations:** ^1^ Department of Pharmacology and Toxicology, Faculty of Medicine Umm Al Qura University Mecca Saudi Arabia; ^2^ Division of Clinical Pharmacology, Department of Laboratory Medicine, Karolinska Institutet Karolinska University Hospital Stockholm Sweden; ^3^ Riksstroke, The Swedish Stroke Registry Region Västerbotten Sweden; ^4^ St Erik Eye Hospital Vitro Retinal Clinic Stockholm Sweden; ^5^ Department of Neurology and Rehabilitation, Faculty of Medicine and Health Örebro University Örebro Sweden; ^6^ Department of Clinical Sciences/Ophthalmology Umeå University Umeå Sweden

**Keywords:** AMD, anti‐VEGF, intravitreal injections, old age, stroke

## Abstract

**Background:**

Intravitreal Anti‐Vascular Endothelial Growth Factor (VEGF) rescues retinal vasculatures and prevents disease progression in patients with neovascular Age‐Related Macular Degeneration (nAMD). However, systemic anti‐VEGF may increase the risk of thromboembolic related complications including stroke and TIA. This study aims to explore the association between stroke and intravitreal anti‐VEGF agents; ranibizumab, aflibercept and bevacizumab.

**Methods:**

This nationwide, population‐ registry‐based case‐control study used registered data 2007–2019. Data from the Swedish Stroke Registry (Riksstroke) and the Swedish Macula Register (SMR) were cross‐linked to identify nAMD patients who developed stroke/TIA within 90 days after intravitreal anti‐VEGF injection. Each stroke case was matched with three controls from Riksstroke with stroke/TIA but no anti‐VEGF treatment.

**Results:**

A total of 33 585 patients with nAMD underwent intravitreal anti‐VEGF agent injections. A stroke occurred in 1693 patients of this group, and 936 of them within 90 days of treatment. Compared with nonuse, intravitreal anti‐VEGF agent use was associated with an increased risk of stroke within 90 days of anti‐VEGF treatment in 2.9% of the nAMD‐patients [Risk Ratio (RR) 1.27, 95% confidence interval (CI) 1.22; 1.33] compared to non‐users. The RR within 30, 31–60 and 61–90 days were 1.36 (1.15; 1.66), 1.40 (1.09; 1.79) and 0.58 (0.52; 0.65), respectively.

**Conclusions:**

Even though the risk is small, intravitreal injections with anti‐VEGF agents for the treatment of nAMD are associated with an increased risk of stroke/TIA. The risk seems to be higher within 60 days of last injection. An assessment of high‐risk populations and risk‐benefit weighting is necessary before intravitreal anti‐VEGF injections are considered.

## 1 INTRODUCTION

Old age is a primary risk factor for vascular diseases, and vascular aging itself is the most prominent risk factor for deterioration of organ function. At the cerebral level, stroke is the leading cause of irreversible disability and death in older people, with an incidence that doubles every 10 successive years after age 55 (Virani et al., 2020). Likewise, in a nearby related organ; the retina, age‐related macular degeneration (AMD) is the leading cause of permanent vision loss in people over age 60; approximately 25% of those ages 60 and over had an early or intermediate AMD, and 2.4% had a late AMD. Not only old age but also other vascular risk factors, for example, hypertension, smoking and inflammation, are associated with both AMD and stroke. Although the potential association is difficult to interrupt and the evidence is still inconclusive (Fernandez et al., 2015), AMD has been claimed to, in itself, increase the risk of stroke (Hu et al., 2010).

Moreover, anti‐vascular endothelial growth factors (anti‐VEGF), the current gold standard treatment of AMD have been reported to be accursed to increase the complex array of stroke. Indeed, anti‐VEGF, when given systemically; intravenously, vascular‐related complications, namely hypertension, myocardial infarction, thromboembolic events and stroke have been identified (Avery et al., 2014; Chen & Cleck, 2009; Kamba & McDonald, 2007).

Owing to their antiangiogenic actions, anti‐VEGF is given systematically for the treatment of patients with various types of cancer. Through the same antiangiogenic actions, intravitreal anti‐VEGF treatment prevents the angiogenesis and increased vascular permeability seen in AMD. Hence, targeting intravitreal VEGF rescues retinal vasculatures and prevents disease progression in patients with neovascular AMD. However, beyond the ocular cavity, systemic VEGF may instead come at the cost of disturbing the microvasculature integrity and the anti‐thrombogenic properties of the endothelium (Enseleit et al., 2010; Tsurumi et al., 1997; Zachary et al., 2000). In this regard, the management of AMD in patients with a high risk of stroke is a challenge where much is still unknown (Kvanta & Lanner, 2016).

From 2006 to date, three anti‐VEGF agents are available for intravitreal injections for nAMD treatment, two of which have specifically gained approval for such indications; ranibizumab (Lucentis; Genentech, South San Francisco, CA) and aflibercept (Eylea; Bayer, Basel, Switzerland and Regeneron Pharmaceutical Inc., Tarrytown, NY, USA). While the third agent, bevacizumab (Avastin; Genentech, Inc., San Francisco, CA, USA), registered for the systemic treatment of various types of solid tumours, is widely used in nAMD in an off‐label manner thanks to its relatively low price as well as its comparable effectiveness to ranibizumab according to some studies (Ferrara et al., 2003; Martin et al., 2011, 2012). Between 2006 and 2011, ranibizumab was the only anti‐VEGF agent used in Sweden. Patients eligible for treatment generally receive serial monthly to 2‐monthly injections and may require these treatments on a regular basis over many years (Sjögren et al., 2017). Noteworthy, AMD is often binocular; patients may require anti‐VEGF injections in both eyes and may thus reach high cumulative exposure.

Although several trials revealed the safety of intravitreal anti‐VEGF therapies, detectable levels of anti‐VEGF agents in the systemic circulation and subsequent prolonged plasma suppression of VEGF after serial intravitreal anti‐VEGF have raised the concern for the occurrence of potential systemic adverse events (Avery et al., 2014; Csaky & Do, 2009; Dedania & Bakri, 2016). Studies that evaluated systemic pharmacokinetics and plasma‐free VEGF levels following intravitreal injections for patients with AMD showed that all three anti‐VEGF agents are rapidly detected in the systemic circulation (Avery et al., 2014; Zehetner et al., 2015). To what extent these detectable levels and these subsequent suppressed effects of intravitreal anti‐VEGF injections may result in such adverse events is still unclear.

As a way of providing insight into this issue, the associations between intravitreal anti‐VEGF injections and the risk of stroke have been investigated; however, the results remain inconclusive. While several studies, including meta‐analyses, have reported no association (Campbell et al., 2012; Chong et al., 2025; Cleary et al., 2011; Dalvin et al., 2019; Etminan et al., 2016; Ng et al., 2015; Zarbin et al., 2018), other studies revealed an increased risk of stroke after intravitreal anti‐VEGF agents (Carneiro et al., 2011; Weinstein et al., 2020). Moreover, in a recent study, the type of stroke has been indicated as well, citing an increased risk of haemorrhagic stroke under a month from discontinuation of ranibizumab exposure, which was possibly owing to the delayed effect of VEGF (Jeon et al., 2021).

No head‐to‐head comparative studies regarding the risk in concern investigating the three anti‐VEGF agents at once have been found, and most of the clinical trials and meta‐analyses of these trials failed to reach the power to detect these rarely occurring adverse events. Thus, this observational study was performed by linking data from the Swedish Macula Register (SMR) and the Swedish Stroke Register (Riksstroke). Both registries are large national population‐based quality registries with excellent coverage, 85%–90% (Macula, 2020; Riksstroke, 2020). The linkage uses the unique personal identification number (PIN) issued to all Swedish residents (Ludvigsson et al., 2009). The study aims to explore if there is an association between stroke and the three intravitreal anti‐VEGF agents: ranibizumab, aflibercept and bevacizumab injections.

## 2 MATERIALS AND METHODS

The study was a nationwide, population‐based registry‐based case–control study using registered data from 2007 to 2019. Data from two Swedish national quality registers were linked via unique personal identity numbers, allowing the study to use near‐complete data covering the Swedish population.

### 2.1 Data sources

The Swedish Stroke Registry (Riksstroke) is a nationwide hospital‐based stroke quality register established in 1994. Since1998, information on comorbidity, procedures, treatment and outcome of patients with acute stroke in all 72 emergency hospitals in Sweden has been registered.

The Swedish Macula Register (SMR) is a national quality registry established in 2003. Since 2008, the registry has become web‐based, collecting data on patients in the active treatment of choroidal neovascularization with intravitreal injections from all eye clinics in Sweden. Neovascular AMD represents 97% of all diagnoses registered in the register. All eye clinics in Sweden that treat nAMD patients participate in the register. Of the treatments provided, 99 percent were anti‐VEGF injections.

### 2.2 Study design and outcome

Data registered in SMR from 2007 to 2019 were cross‐linked with data in Riksstroke, to identify patients with nAMD who developed stroke/TIA within 30, 31–60 and 61–90 days after receiving the last intravitreal Anti‐VEGF injection. Information on Anti‐VEGF treatment taken by the patient, including agent name and number of injections, was obtained from SMR. The time in days from the first and last Anti‐VEGF injection to the development of stroke events, information on vascular risk factors including age, smoking, the presence or absence of diabetes, treatment with antihypertensive agents (as a proxy for hypertension), previous TIA and atrial fibrillation were obtained from the Riksstroke. Each stroke case was matched by age, sex and stroke diagnosis with three controls from the Riksstroke without previous Anti‐VEGF treatment.

### 2.3 Statistical analysis

Statistical analysis was conducted in R (R‐Core‐Team, 2020) with a statistically significant level set to *p* < 0.05. The continuous variable was described using mean values and standard deviation. Count data were summarized in frequencies and percentages, and Fisher exact test was used to test for equal proportions between patient groups. Adjusted analysis was conducted using multiple Poisson regression. Results from multiple regression are presented as adjusted relative risk (RR) with 95% confidence intervals (CI).

### 2.4 Ethical considerations

The study was approved by the Swedish Ethical Review Authority (reference no. 2018/89–31/1). In accordance with the Personal Data Act (Swedish law No. SFS 1998:204), no informed consent is needed to collect data from medical charts and inpatient records for quality registers, but patients are informed of the possibility of opt‐out.

## 3 RESULTS

### 3.1 Number of patients

**FIGURE 1:**
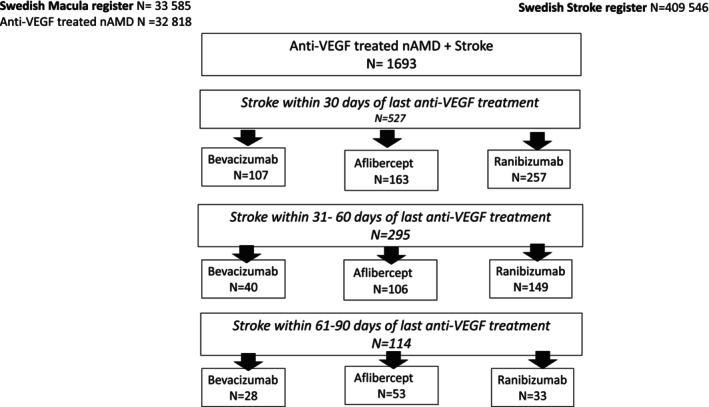
Number of patients in Riksstroke and SMR with a stroke event within 30 days, 31–60 days and 61–90 days of the last intravitreal anti‐VEGF treatment.

Riksstroke has 409 546 stroke patients, while SMR has 33 585 nAMD patients who have undergone intravitreal anti‐VEGF injections between 2007 and 2019. Of the total number of injections, 26 263 were unilateral and 6555 were bilateral treatment. By cross‐linking the two databases, we identified that 1693 patients had a stroke; among these patients, 1330 received ranibizumab, 503 received aflibercept and 314 received bevacizumab. Of all stroke incidents, 936 occurred within 90 days of the last treatment, 527 within 30 days, 295 within 31–60 days and 114 within 61–90 days (Figure 1).

Compared with non‐use, intravitreal anti‐VEGF agent use was associated with an increased risk of stroke within 90 days of the last anti‐VEGF treatment in 2.9% of the nAMD patients [Risk Ratio (RR) 1.27, 95% confidence interval (CI) 1.22; 1.33] compared to non‐users. The incidence of stroke within 90 days of the last anti‐VEGF treatment was 2.5% (439) for ranibizumab, 1.9% (322) aflibercept and 2.9% (175) for bevacizumab. The risk was higher for those treated with aflibercept, RR 1.36 (1.23–1.52) and bevacizumab, RR 1.26 (1.10–1.46) Table 1.

**TABLE 1:** Incidence of stroke within 90 days of last Anti‐VEGF treatment in SMR with Ranibizumab, Aflibercept and Bevacizumab.

	Ranibizumab, *n* = 17 670	Aflibercept, *n* = 16 624	Bevacizumab, *n* = 8323	Total, *n* = 32 818
Number (%)	439 (2.5%)	322 (1.9%)	175 (2.1%)	936 (2.9%)
RR crude	0.92 (0.85;1.00)	1.36 (1.23;1.52)	1.26 (1.10;1.46)	1.27 (1.22;1.33)

*Note*: Aflibercept and Bevacizumab.

The risk for stroke decreased with time and after more than 60 days of the last injection the risk was lower for all anti‐VEGF treatment (Tables 2 and 3).

**TABLE 2:** Incidence of stroke within 30, 31–60 and 61–90 days of last treatment in SMR with Anti‐VEGF.

	Anti‐VEGF total *N* = 32 818		
	Days after last injection		
	0–30 days	31–60 days	61–90 days
Number (%)	527 (1.6%)	295 (1.0%)	114 (0.3%)
RR crude	1.38 (1.15;1.66)	1.40 (1.09;1.79)	0.58 80.52;0.65)

**TABLE 3:** Incidence of stroke within 30, 31–60 and 61–90 days of last Anti‐VEGF treatment in SMR with Ranibizumab, Aflibercept and Bevacizumab.

	Ranibizumab, *n* = 17 670		Aflibercept, *n* = 16 624		Bevacizumab, *n* = 8323	
	RR	95% CI	RR	95% CI	RR	95% CI
1 to 30 days	1.02	0.76;1.34	1.88	1.54; 2.27	1.40	1.07; 1.80
31 to 60 days	1.00	0.78;1.27	1.68	1.38; 2.03	1.21	0.92; 1.56
61 to 90 days	0.50	0.27;0.85	0.56	0.48; 0.67	0.71	0.57; 0.89

### 3.2 Comparison between cases and controls

Comparison of patient characteristics between those who developed stroke within 90 days of last treatment and all controls, that is, stroke patients without previous anti‐VEGF treatment, showed no difference in presence of hypertension (measured as using antihypertensive medicines), atrial fibrillation, a previous TIA or reported smoking (Table 4).

**TABLE 4:** RR for a stroke within 90 days for stroke patients on anti‐VEGF treatment and Non anti‐VEGF treatment.

Characteristic	Anti‐VEGF treatment, *N* = 1693			Non anti‐VEGF treatment, *N* = 109		
	RR	95% CI^a^	*p*	RR	95% CI^a^	*p*
Smoker	1.01	0.99, 1.03	0.4	0.95	0.82, 1.20	0.6
Antihypertensiv treatment	1.01	0.93, 1.10	0.9	0.94	0.34, 2.59	0.9
Previous stroke	1.04	0.95, 1.16	0.4	0.72	0.34, 1.77	0.4
Previous TIA	0.98	0.94, 1.03	0.3	0.81	0.38, 2.26	0.6
Arterial fibrilation	1.00	0.94, 1,07	0.8	0.91	0.24, 3.01	0.9
Diabetic	0.96	0.91, 1.03	0.2	0.85	0.40, 2.43	0.7

### 3.3 Number of injections

A stroke patient received a mean of 21.0 (SD ± 1.0) injections, slightly more frequent than patients who did not develop a stroke, who received a mean of 19.4 (SD ± 0.2) injections. Most patients in both groups were treated with ranibizumab (Table 5).

**TABLE 5:** Mean number of injections for patients with anti‐VEGF treatment without development of stroke and patients who developed stroke.

	Patients with anti‐VEGF treatment without development of stroke (*n* = 31 892)	Patients with anti‐VEGF treatment who developed stroke (*n* = 1693)
Anti‐VEGF	19.4 ± 0.2	21.0 ± 1.0
Ranibizumab	24.9 ± 0.4	23.0 ± 1.3
Aflibercept	12.2 ± 0.2	12.9 ± 1.4
Bevacizumab	17.5 ± 0.4	18.8 ± 2.9

*Note*: mean ± SD.

## 4 DISCUSSION

When systematically administered, anti‐VEGF agents have been reported to cause hypertension, myocardial infarction, thromboembolic events and stroke (Ranpura et al., 2010; Skillings et al., 2005; Tlemsani et al., 2016). In a pooled analysis of data from 16 trials in which patients were treated with or without bevacizumab in combination with concurrent standard antineoplastic therapy, intravenous bevacizumab is significantly associated with a 3.2‐fold increased risk of cerebrovascular events (Zuo et al., 2014).

In this study, 2.9% of the nAMD patients treated with intravitreal anti‐VEGF agents had a stroke within 90 days of their last anti‐VEGF treatment. The risk of stroke declined after 60 days. During the study period, the overall incidence of stroke decreased. A Swedish study showed that the incidence of stroke between 2005 and 2018 decreased in both men (from 293 to 223 per 100 000) and women (from 278 to 191 per 100 000) (Eriksson et al., 2021). The stroke incident in patients with anti‐VEGF treatment reported in this study falls between previously published results that were either lower or higher. A lower incidence of stroke has been reported in a meta‐analysis with odds ratio for stroke outcome calculated to be 0.66 (CI; 0.34–1.28) and 0.85 (CI; 0.3–2.4) (*p* = 0.78) in RCTs comparing bevacizumab–ranibizumab (*n* = 3093) and ranibizumab–aflibercept (*n* = 2419) in patients with nAMD (Sjögren et al., 2017). This meta‐analysis was conducted in order to study the efficacy and safety of anti‐VEGF treatment of various macula diseases. The safety outcome has been measured by extracting spontaneous reports of the adverse effects from respective randomized trials. These results are in line with that pointed out in another meta‐analysis with 2809 patients where the estimated RR of stroke (four trials) and TIA (three trials) following treatment with bevacizumab versus ranibizumab by 1 year were 0.64 (0.21–1.95) and 1.02 (0.26–4.07) (Solomon et al., 2016).

Indeed, a much higher incidence has even been reported. Through comparing the thromboembolic events during 2 years prior to and 2 years following the initial intravitreal bevacizumab injection in 2102 nAMD patients, stroke rates were higher 2 years after treatment (*p* = 0.01) with odds ratios for stroke during the 2 years prior versus the 2 years after being 1.21 (95% CI, 0.71–2.06) and 2.70 (95% CI 1.33–5.50) respectively (Weinstein et al., 2020).

Moreover, based on pooling data from three RCTs in which 859 subjects were treated with monthly ranibizumab and 434 subjects were sham‐treated during the 2‐year observation period, 19 of the ranibizumab‐treated subjects (2.2%) were found to have cerebrovascular accidents, whereas 3 (0.7%) of the sham‐treated subjects were thereby intravitreal ranibizumab was associated with the incidence of cerebrovascular accidents by 3.2‐fold (95% CI, 0.96–10.95; *p* < 0.045) (Ueta et al., 2009).

However, due to relatively small pooled data (Chakravarthy et al., 2012; Martin et al., 2012; Schmidt‐Erfurth et al., 2014), more than half of the patients were lost at an early stage of the trial (Berg et al., 2017), influencing sponsor expectation (Antoszyk et al., 2008; Ueta et al., 2009) along with the potential inadequacy of pooling available trials and variants in terms of population, interventions and outcomes, the findings of these studies need to be interpreted with caution, especially when bearing in mind that these analyses do not consider specific rare events, it is difficult to detect any significant differences between the analyses (Csaky & Do, 2009).

Nevertheless, in a large observational cohort study conducted on the scale of the entire French population, the results showed no increase in stroke risk with ranibizumab (*n* = 174 794) and aflibercept (*n* = 76 242), (*n* = 2306 incident strokes, a HR 1.03; 95% CI 0.95–1.13) (Billioti de Gage et al., 2022). Albeit, this study found a trend to an increased risk of stroke in new users of aflibercept compared to those using ranibizumab among patients with diabetes (Billioti de Gage et al., 2022).

Another significant aspect of our study is that in the risk for stroke, the difference in RR between ranibizumab and aflibercept and bevacizumab shows a lower RR for ranibizumab. The tendency of a lower incidence in the ranibizumab group compared to the bevacizumab group is comparable to that found in several studies: (0.913% vs. 6.186%) (Carneiro et al., 2011), (9.2% vs. 12.3%) (Krebs et al., 2013), (9.6% vs. 12.5%) (Chakravarthy et al., 2013) and (20.5% vs. 25.7%) (Martin et al., 2012) respectively. As a result of its transient appearance in the systemic circulation and rapid clearance, ranibizumab is not as systemically effective as bevacizumab and aflibercept and thereby has an insignificant effect on plasma‐free VEGF levels. The latter two agents appear to accumulate with repeated administration and produce a marked reduction in plasma‐free VEGF, posing the possibility of systemic side effects (Avery et al., 2014; Matsuyama et al., 2010; Zarbin, 2018). Unfortunately, it is unknown how much anti‐VEGF agent reaches the general circulation after being injected intravitreal. Nevertheless, a study found that only small doses of bevacizumab, such as 1.25 mg/0.05 mL of a single injection, significantly reduced levels of VEGF in the blood; almost 23% of baseline for at least a month after administration (Matsuyama et al., 2010).

With regard to ranibizumab compared with aflibercept, in a retrospective study of 12 215 nAMD patients newly treated with intravitreal aflibercept or ranibizumab and followed up for 2 years, or until the occurrence of thromboembolic events, including ischemic heart disease, ischemic stroke, transient ischemic attack, deep vein thrombosis and pulmonary embolism, death or the end of the study period, patients, aflibercept was associated with a lower risk of thromboembolic events (adjusted hazard ratio 0.85; 95% CI 0.77–0.94) (Lee et al., 2022). The result from this later study is, however, much lower than our own (RR of 1.36), which showed a greater risk associated with aflibercept. Interestingly, aflibercept has been reported to reduce plasma‐free VEGF the most, which may be due to its higher affinity for VEGF (Avery et al., 2014; Papadopoulos et al., 2012), the fact that mirrors our finding as the risk associated with aflibercept was significantly higher than with other agents.

However, these variations in stroke risk between the agents in our study need to be interpreted with caution as our data were not able to determine whether they were caused by confounding by indication or by an actual risk difference. The majority of our study cases (78.5%) were treated with ranibizumab, which could be partially explained by the fact that ranibizumab was the only anti‐VEGF agent used in Sweden between 2006 and 2011.

The most prevalent risk factors for stroke are registered in Riksstroke. There were no significant differences between the study population and controls RR for the different risk factors registered in Riksstroke.

Since the cerebrovascular system is compromised following a stroke, angiogenesis is expected to play an even more prominent role in tissue remodelling. By blocking angiogenesis, anti‐VEGF might have a detrimental effect. Studies that determined the systemic safety of intravitreal anti‐VEGF among those who had a previous stroke were primarily based on investigating the mortality outcome. A cohort study revealed a significantly higher mortality rate among the nAMD patients with prior stroke/AMI (adjusted HR = 2.37; 95% CI, 2.14–2.62) (Chen et al., 2021). Another study comparing mortality over time in patients treated with bevacizumab for AMD compared to patients for whom there was no record of a prescription for an anti‐VEGF agent found increased mortality within 3 months after a cerebrovascular event (OR = 6.92, 95%, CI 1.88–25.43, *p* < 0.01), within 6 months (OR = 2.00, 95%, CI 0.96–4.16, *p* = 0.064) while the risk was insignificant within 12 months (OR = 1.30, 95%, CI 0.75–2.26, *p* = 0.348) (Hanhart et al., 2018). As noted, these studies measured the mortality rather than the incidence of the stroke itself, and they chose to study the all‐cause mortality; the incidence of death, without identifying the direct cause of death. Since there were other causes of death besides cerebrovascular events, this could potentially be a concern when interpreting the results, particularly when looking at this age group of patients where heart diseases, cancer, infections and Alzheimer's disease also occur frequently. Though their findings reflect those of our results and are necessary for determining the eligibility for treatment, whether patients after cerebrovascular events should be regarded as a high‐risk population and therefore, precautions are considered before deciding on treatment with intravitreal anti‐VEGF injections.

Atrial fibrillation is another well‐known risk factor for stroke. During the last decade, the proportion of the Swedish population with atrial fibrillation treated with anticoagulants has increased and is now over 80% (Loikas et al., 2017). When our study started, there was still an under treatment, particularly among women, that may have influenced the risk (Forslund et al., 2018). Hypertension, in our study defined as being on antihypertensive treatment at the time of stroke, also increase the risk of stroke. As with atrial fibrillation, the presence of hypertension indicates a higher stroke risk. The use of antihypertensive agents is expected in this age group. If it is hypertension by itself, the treatment or even a particular drug class of antihypertensive treatment cannot be elucidated by our data. However, as far as we know, there are no data on interactions between antihypertensive agents and anti‐VEGF agents. Smoking has been shown to be a risk factor not only for stroke but also for nAMD, both with early and late onset (Wang et al., 2022). Smoking is the one lifestyle factor we analysed and we did not find a significant difference. However, the material is not that large, and we only have information on smoking status in the patients with stroke, not in those without, which is a limitation.

As of yet, the evidence in the literature regarding the association of anti‐VEGF treatment with stroke revealed no consensus. The clinical trials and meta‐analyses are designed to examine mainly the efficacy, rather than low frequency systemic serious adverse reactions following intravitreal injection of an anti‐VEGF agent. In such a dilemma, safety profile and risk assessment will be achieved only through post‐marketing surveillance or through analyses of data from healthcare databases. Using data from two large national quality registers, linking the patients with the unique PIN, enables us to have an extensive data set including more than 30 000 patients with nAMD who underwent intravitreal anti‐VEGF injections. The validity of the diagnoses and treatments has been shown to be high in Riksstroke and SMR. The incidence of stroke after overall anti‐VEGF use has not been reported as far as we know. The studies reporting such incidents mainly compare two agents, such as ranibizumab and bevacizumab or ranibizumab and aflibercept; no comparative studies have been performed investigating all three anti‐VEGF drugs simultaneously. In this study, stroke incidence was calculated based on data collected simultaneously for the three anti‐VEGF agents available for the treatment of nAMD. As in all observational studies, confounding by indication cannot be excluded.

## 5 CONCLUSION

Findings from this large pharmaco‐epidemiologic study suggest that intravitreal injections with anti‐VEGF agents for the treatment of nAMD are associated with a small increase in the risk of stroke. The risk seems to be higher for aflibercept and bevacizumab than with ranibizumab. The risk for stroke decreases after 60 days since the last anti‐VEGF injection. For patients with nAMD in need of anti‐VEGF treatment who have a history of cerebrovascular events, have atrial fibrillation or hypertension, and who are smokers, it seems prudent to try to reduce stroke risk factors.

## FUNDING INFORMATION

This study was supported by a grant from Region Stockholm, Sweden.

## CONFLICT OF INTEREST STATEMENT

AF, KSL, FJ, SAS and MvE declare no conflicts of interest. IW is an advisory board member for Abbvie, Bayer and Roche.

## DISCLAIMER

Karin Söderberg‐Löfdal is employed at the Swedish Medical Products Agency, SE‐751 03 Uppsala, Sweden; the views expressed in this paper are the personal views of the authors and not necessarily the views of the Government agency.
